# Plant extract enhances the viability of *Lactobacillus delbrueckii* subsp*. bulgaricus* and *Lactobacillus acidophilus* in probiotic nonfat yogurt

**DOI:** 10.1002/fsn3.189

**Published:** 2014-12-12

**Authors:** Minto Michael, Randall K Phebus, Karen A Schmidt

**Affiliations:** Food Science Institute, Kansas State UniversityManhattan, Kansas, 66506

**Keywords:** *Bifidobacterium animalis*, buffering capacity, *Lactobacillus acidophilus*, plant extract, probiotics, yogurt

## Abstract

A commercial plant extract (prepared from olive, garlic, onion and citrus extracts with sodium acetate (SA) as a carrier) was evaluated to extend the viability of yogurt starter and probiotic bacteria as a means to enhance the shelf life of live and active culture, probiotic nonfat yogurt. Yogurts prepared from three different formulas (0.5* plant extract, 0.25* SA, or no supplement) and cultures (yogurt starter plus *Bifidobacterium animalis*,*Lactobacillus acidophilus*, or both probiotics) were assessed weekly during 29 days of storage at 5°C. Supplemented yogurt mixes had greater buffering capacities than non-supplemented yogurt mixes. At the end of storage, *Lactobacillus bulgaricus* and *L. acidophilus* counts in supplemented yogurts were greater compared with non-supplemented yogurts. Supplementation did not affect *Streptococcus thermophilus* and *B. animalis* counts. Hence the greater buffering capacity of yogurt containing plant extract could enhance the longevity of the probiotics, *L. bulgaricus* and *L. acidophilus*, during storage.

## Introduction

The FAO/WHO defines probiotics as live microorganisms, which when consumed in adequate amounts, confer health benefits to the host (Moriya et al. 2006; Vasiljevic and Shah 2008). General agreement has not been reached concerning the minimum concentration of live probiotic bacteria at the time of consumption to confer health benefits (Donkor et al. 2006); however, the recommended concentrations in foods range from 6 to 8 log cfu/g (Ross et al. 2005; Vasiljevic and Shah 2008). The market for probiotic foods has grown rapidly, and yogurt is one of the most popular vehicles for consuming probiotics (Stanton et al. 2001).

The viability of probiotic bacteria in yogurt often decreases below the recommended concentration (6 log cfu/g) during storage because of low pH, high oxygen tension, increased redox potential (Eh) and increased hydrogen peroxide concentration (Dave and Shah 1997a,b; Lourens-Hattingh and Viljoen 2001; Donkor et al. 2006; Vasiljevic et al. 2007; Sarkar 2008). Some probiotic bacteria, especially *Bifidobacterium* spp., are sensitive to low pH (<4.5), and their viability in yogurt decreases rapidly (in some cases within a week) during storage depending upon the strain (Shah et al. 1995; Lourens-Hattingh and Viljoen 2001). Greater buffering ability in yogurt may counter-act the lethal effect of the acidic environment on starter and probiotic bacteria, and extend the life of these bacteria (Ainaz and Ehsani 2008; Shafiee et al. 2010).

The National Yogurt Association (NYA) is a U.S. national non-profit organization representing manufacturers and marketers of live-active culture yogurt products and suppliers to the yogurt industry, with the aim of sponsoring research for live-active culture yogurt and providing an information resource to the public (NYA 2012). In the U.S., NYA has established a voluntary program according to which, refrigerated yogurts displaying the “Live & Active Cultures” seal on the containers should have ≥8 log cfu/g yogurt bacteria (i.e., sum of *Streptococcus thermophilus* and *Lactobacillus bulgaricus* counts) at the time of manufacture (NYA 2012). Yogurts with the “Live & Active Cultures” seal should also pass the “culture activity test” at the end of shelf life, that is, rehydrated and pasteurized 12* nonfat dry milk should have an increase of ≥1 log cfu/g in total yogurt bacteria counts when inoculated with 3* yogurt sample and fermented at 43°C for 4 h (NYA (National Yogurt Association) 2012). Generally, on a quality basis, yogurt has a shelf life of 4–7 weeks (Chandan and O'rell 2006); however, yogurts with live-active cultures should have individual counts of each bacteria ≥6 log cfu/g until the end of stated shelf life (Lourens-Hattingh and Viljoen 2001).

Sodium acetate (SA) is a U.S. Food and Drug Administration (FDA)-approved buffering and flavoring agent (Lindsay 2007; Manju et al. 2007). Although no published studies address the effect of SA on the viability of starter and probiotic bacteria in yogurt, researchers have reported that the growth yield and acid-producing ability of some lactic acid bacteria are enhanced when grown in media supplemented with SA (Lino et al. 2001, 2002). Cegemett ® Fresh (Cognis, Nutrition & Health, Monheim, Germany) is marketed as an antimicrobial and antioxidant plant extract (PE) prepared from an oleoresin mixture (olive, garlic, onion, citrus extract and ascorbic acid) with SA as a carrier (Heller 2007). Michael et al. (2010) reported that Eh (a measure of antioxidant ability) did not differ in PE-supplemented and non-supplemented (NS) yogurts (∼375 mV), but *Lactobacillus delbrueckii* subsp*. bulgaricus* counts in yogurts supplemented with 0.5 and 1.0* PE were >6 log cfu/mL for an additional 21 and 14 days, respectively, compared with the NS yogurt. Michael et al. 2010 concluded that factors (such as buffering capacity) other than Eh were responsible for the improved longevity of *L. bulgaricus*. If the enhanced buffering ability of PE-supplemented yogurts was responsible for the improved *L. bulgaricus* viability, the PE supplementation may also be advantageous for probiotic bacteria.

The objective of this study was to investigate if the adjustment of the buffering capacity of yogurt mix (by supplementing yogurt mix with PE) could increase the longevity of yogurt starter (*S. thermophilus* and *Lactobacillus delbrueckii* subsp. *bulgaricus*) and probiotic (*Bifidobacterium animalis* subsp. *animalis* and *Lactobacillus acidophilus*) bacteria, hence increase the shelf live of live-active culture probiotic nonfat yogurt, during 29 days of storage at 5°C.

## Materials and Methods

### Experimental design

Yogurt mixes were formulated with 0.5* (w/v) PE (Cegemett ® Fresh), 0.25* (w/v) SA (Fisher Biotech, Fisher Scientific, Fair Lawn, NJ), or no supplement (NS). Sodium acetate supplementation was used as a comparison treatment because PE contains SA as a carrier (concentration of SA in PA was not disclosed by the manufacturer), and SA has been reported to increase the growth yield of some lactic acid bacteria (Lino et al. 2001, 2002). Each yogurt mix formulation was fermented with yogurt starter cultures plus *B. animalis* (B), *L. acidophilus* (L), or both probiotics (P). Yogurts were manufactured, stored at 5°C for 29 days, and analyzed weekly. Three replications were conducted, and each analysis was done in duplicate and the average was used for statistical analysis.

A repeated measure (storage) analysis in a 3 × 3 (formula × culture) factorial randomized complete block design with fixed blocks (replications) was used for statistical analysis. Analysis of variance (ANOVA) and least square means at *α *= 0.05 were used to identify and differentiate means of the significant main effects and interactions. All analyses were performed using the PROC MIXED procedures of SAS version 9.3 (SAS ® Institute Inc., Cary, NC).

### Yogurt starter and probiotic cultures propagation

Freeze-dried yogurt culture (Yo-Mix Yogurt Cultures, Yo-Mix 161 LYO 375 DCU, Danisco, New Century, KS) was propagated as described by Michael et al. (2010), and maintained at 5°C until used as the mother culture in yogurt (within 48 h). *Bifidobacterium animalis* subsp. *animalis* ATCC 25527 (American Type Culture Collection, Manassas, VA) and *L. acidophilus* ATCC 4356 (Microbiologics, St. Cloud, MN) cultures were initially propagated according to supplier's instructions. Nonfat dry milk (NFDM; low heat, spray processed, Grade A, Dairy America ™, Fresno, CA) was rehydrated at 140 g/L in distilled-deionized water, supplemented with 1 g glucose (Fisher Scientific) and 1 g yeast extract (Acros Organic, Fisher Scientific), sterilized at 121°C and 105 kPa for 15 min, and cooled to 37°C. Sterilized, reconstituted NFDM was inoculated with 3* (w/w) *B. animalis* or *L. acidophilus* culture, incubated at 37°C for 18 h, and maintained at 5°C until used as the mother culture in yogurt (within 48 h). For *B. animalis* propagation, sterilized, supplemented reconstituted NFDM (90 mL) was also supplemented with 10 mL 0.5* l-cysteine. HCl (Fisher Biotech, Fisher Scientific) solution.

### Yogurt preparation

Set style yogurt samples were prepared as described by Michael et al. (2010); but with an incubation temperature of 40°C, and addition of *B. animalis*,*L. acidophilus* or both probiotics along with the yogurt starter cultures during fermentation.

### Titratable acidity and pH

Titratable acidity (expressed as the percentage of lactic acid) and pH were measured as described by Michael et al. (2010).

### Buffering capacity/curves

Buffering capacity was measured at 25°C as described by Salaün et al. (2007) with some modifications. Acid titration was performed on 10 mL yogurt mix from initial pH to 4.00 by using 1N hydrochloric acid (Fisher Scientific) added in 0.05 mL increments at 30 sec intervals. Buffering capacities were calculated using the formula described by van Slyke (1922) and plotted against the corresponding pH values to generate buffering curves. The buffering curves of NS, PE and SA yogurt mixes from all replications were plotted, and the curves best representing the average of all replications for each yogurt mix were selected and used for interpretation. The following formula was used to calculate buffering capacity:

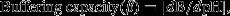
1where, dB = mL of acid added/mL of sample.

dpH = pH after adding acid - pH before adding acid.

### Microbial counts

*Streptococcus thermophilus* and *L. bulgaricus* counts were enumerated, and confirmed as described by Michael et al. (2010). *Bifidobacterium animalis* counts were enumerated as described by Moriya et al. (2006) with some modifications. MRS agar was prepared and tempered to 45°C in a water bath (Precision model 183; Precision Scientific, Chicago, IL). A supplement solution consisting of l-cysteine. HCl (0.5 g), nalidixic acid (15 mg), neomycin sulfate (100 mg), lithium chloride (3 g) and paromomycin sulphate (200 mg) dissolved in 40 mL distilled-deionized water was prepared (all chemicals obtained from Fisher Scientific). The supplement solution was filter sterilized through a 0.42 *μ*m pore membrane (Fisher Scientific), and 4 mL of supplement solution was mixed with 96 mL of the tempered agar just before plating. Yogurt samples were serially diluted using sterilized 0.1* peptone water, pour plated using supplemented MRS agar, and incubated anaerobically using anaerobe gas packs at 37°C for 72 h. *Bifidobacterium animalis* colonies were confirmed using Gram staining and the API 20 A system (bioMérieux, Inc., Durham, NC).

*Lactobacillus acidophilus* counts were enumerated as described by Dave and Shah (1996) with some modifications. Sterilized and tempered (45°C) MRS agar (90 mL) was supplemented with filter-sterilized d-sorbitol solution (10 mL), prepared by dissolving 10 g d-sorbitol (Fisher Scientific) in 100 mL of distilled-deionized water, just before plating. Yogurt samples were serially diluted using sterilized 0.1* peptone water, pour plated using supplemented MRS agar, and incubated anaerobically using anaerobe gas packs at 37°C for 72 h. *Lactobacillus acidophilus* colonies were confirmed using Gram staining and the API 20 A system (bioMérieux, Inc.).

## Results and Discussion

### Buffering capacity/curves

**Figure 1:**
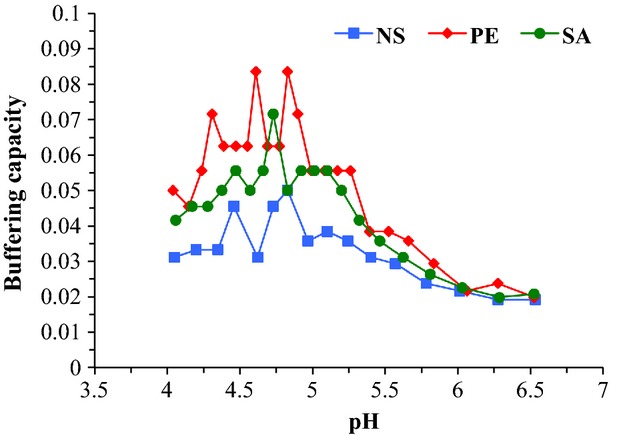
Buffering curves of non-supplemented (NS), plant extract (PE) supplemented and sodium acetate (SA) supplemented yogurt mixes.

Buffering curves for NS and supplemented yogurt mixes are presented in Figure1. Overall, buffering capacities of the PE and SA yogurt mixes were greater than that of the NS yogurt mix at pH <6; however, buffering capacity of the PE yogurt mix was greater than that of the SA yogurt mix (except at pH 4.73). Non-supplemented and SA yogurt mixes had maximum buffering capacity (exhibited as peaks; 0.050 and 0.071, respectively) at pH 4.83 and 4.73, respectively. Buffering compounds exhibit maximum buffering capacity at the pH equal to their pKa (van Slyke 1922), and the pKa value for SA is 4.76 (Ruzin 1999); therefore, the greater buffering action of SA yogurt mix compared with NS yogurt mix could be attributed to the presence of SA. Yogurt supplemented with PE had two buffering capacity peaks (0.083), one at pH 4.83 and the second at pH 4.61. These results indicated that there are some factors/ingredients in PE, other than SA, which contributed to the greater buffering capacity of PE yogurt mix. Greater buffering capacity of yogurt mix during fermentation and storage can counteract the lethal affect of pH on the viability of yogurt and probiotic bacteria by slowing the pH decline despite the increase in lactic acid in yogurt. Shafiee et al. (2010) reported that greater buffering capacities of yogurt mixes, due to greater milk solids not fat (8* and 12*), had greater total Bifidobacteria and *L. acidophilus* counts (6.18–7.61 log cfu/mL) at the end of the fermentation compared with yogurt mixes with 4* milk solids not fat (5.84–6.95 log cfu/mL), which had lower buffering capacities. Ainaz and Ehsani (2008) reported similar results for probiotic yogurts during storage. They concluded that yogurt mixes with greater protein contents (4.28–6.80*) demonstrated greater buffering capacities, and had greater total *B. lactis* and *L. acidophilus* counts during the 21 days of storage compared with yogurt mix with 3.06* protein content, which had a lower buffering capacity.

Maximum buffering capacity of raw milk during acid titration has been reported to occur at ∼ pH 5.1 (Lucey et al. 1993b), whereas milk that has been heat-treated at 90°C for 10 min had maximum buffering capacity at ∼ pH 5.0 (Lucey et al. 1993a). The difference between the pH for maximum buffering capacity in the Lucey et al. (1993a) study and for the NS yogurt mix in this study could be due to differences in total solids. Gastaldi et al. (1997) reported that buffering capacity increased in reconstituted skim milk when total solids increased from 10* (∼0.038) to 15* (∼0.062) and 20* (∼0.085). They also reported that pH for maximum buffering capacity of reconstituted skim milk decreased when total solids increased from 10* (∼pH 5.0) to 15* and 20* (∼pH 4.8).

### Titratable acidity and pH

Yogurt pH and TA were significantly affected by formula and storage (Table1). Yogurt pH was also affected by formula × culture; whereas, yogurt TA was affected by culture only (Table1). The pH of PE yogurts (4.38) was highest, followed by SA yogurts (4.34) and NS yogurts (4.27); whereas, the TA of PE yogurts (1.48*) was also highest, followed by SA yogurts (1.37*) and NS yogurts (1.23*; Table2). These results confirmed that PE yogurts had greater buffering ability compared with the SA and NS yogurts. Therefore, some additional factors, other than SA in the PE, contributed to the greater buffering ability of PE yogurts compared with SA yogurts.

**Table 1:** *P*-values of the main effects and interaction effects for various parameters during storage.

	*P*-value					
Effect	pH	TA	*Streptococcus thermophilus*	*Lactobacillus bulgaricus*	*Bifidobacterium animalis*	*Lactobacilluss acidophilus*
Formula	<0.0001^1^	<0.0001^1^	0.6724	<0.0001^1^	0.0092^1^	0.0116^1^
Culture	0.4356	0.0499^1^	0.5230	0.2281	0.8059	0.3566
Storage	<0.0001^1^	<0.0001^1^	<0.0001^1^	<0.0001^1^	<0.0001^1^	<0.0001^1^
Formula × culture	0.0461^1^	0.3411	0.6254	0.0157^1^	0.2119	0.4288
Formula × storage	0.5098	0.9349	0.499	<0.0001^1^	0.3178	0.0203^1^
Culture × storage	0.9345	0.4264	0.9258	0.0235^1^	0.9559	0.1568
Formula × culture × storage	0.9158	0.4911	0.8726	0.5542	0.6881	0.6119

1 Main and/or interaction effect was significant (*P *≤* *0.05).

**Table 2:** pH and titratable acidity (TA), and *Lactobacillus bulgaricus*,*Bifidobacterium animalis* and *Lactobacillus acidophilus* counts (log cfu/mL) of stored yogurts as a function of formula.

	Formula			
Parameter	NS	PE	SA	Pooled SE
pH	4.27^c^	4.38^a^	4.34^b^	0.01
TA (* lactic acid)	1.23^c^	1.48^a^	1.37^b^	0.02
*L. bulgaricus*	6.66^c^	7.68^a^	7.24^b^	0.11
*B. animalis*	5.67^ab^	5.34^b^	6.05^a^	0.13
*L. acidophilus*	6.94^b^	7.52^a^	7.21^ab^	0.11

Means (*n* = 45; average for culture and storage days) with different superscripts within a row are different (*P *≤* *0.05). NS, non-supplemented yogurts; PE, plant extract supplemented yogurts; SA, sodium acetate supplemented yogurts.

**Figure 2:**
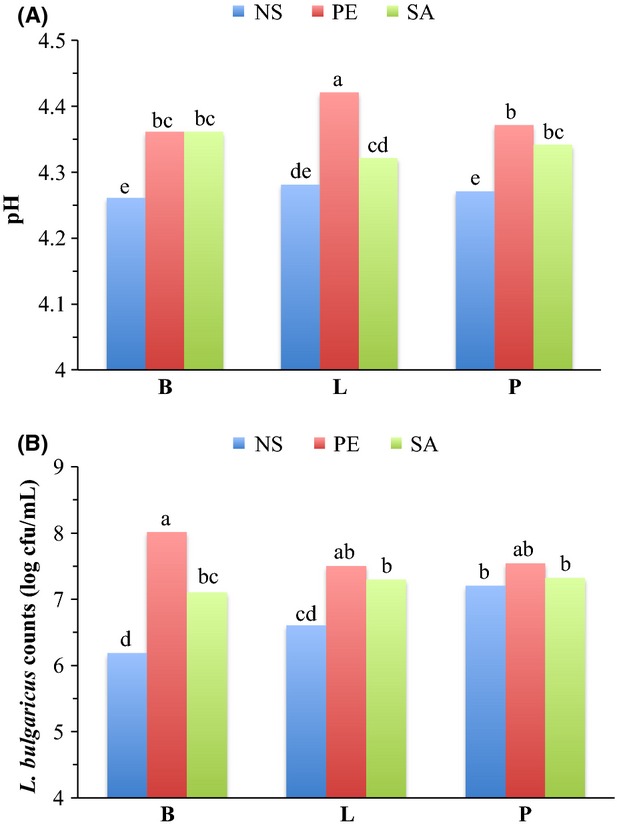
pH (A) and *Lactobacillus Bulgaricus* counts (B) of stored yogurts^x^ as a function of formula × culture. (A) means (*N* = 15) averaged for storage days with pooled standard error of 0.02; (B) means (*N* = 15) averaged for storage days with pooled standard error of 0.19. NS, non-supplemented yogurts; PE, plant extract supplemented yogurts; SA, sodium acetate supplemented yogurts; B, yogurts fermented with *Bifidobacterium animalis*; L, yogurts fermented with *Lactobacillus acidophilus*; P, yogurts fermented with *B. animalis* and *L. acidophilus*. Bars with different letters are different (*P *≤* *0.05)

Yogurt pH was not affected by the culture; however, the TA of yogurts fermented with P culture (1.39*) was greater compared with the TA of yogurts fermented with L culture (1.33*; Table3). The TA of yogurts fermented with B culture (1.37*) was similar to the yogurts fermented with L or P culture (Table3). During storage, yogurt pH significantly decreased from day 1 (4.42) through day 15 (4.31), and then remained constant until day 29 (4.29); whereas, yogurt TA significantly increased from day 1 (1.31*) through day 15 (1.37*), and then remained constant until day 29 (1.39*; Table4). Mani-López et al. (2014) reported the similar trend of decrease in pH and increase in TA during 35 days of storage in probiotic yogurt fermented with yogurt cultures and *L. acidophilus, L. casei* or *L. reuteri*. The pH of PE yogurt fermented with L culture (4.42) was highest and the pH of NS yogurt fermented with B or P culture (4.26 or 4.27, respectively) was lowest (Fig.2A). The pH within NS or SA yogurts fermented with different cultures was similar (Fig.2A).

**Table 3:** Titratable acidity (TA) of stored yogurts as a function of culture.

	Culture			
Parameter	B	L	P	Pooled SE
TA (* lactic acid)	1.37^ab^	1.33^b^	1.39^a^	0.02

Means (*n* = 45; average for formula and storage days) with different superscripts within the row are different (*P *≤* *0.05). B, yogurts fermented with *B. animalis*; L, yogurts fermented with *L. acidophilus*; P, yogurts fermented with *B. animalis* and *L. acidophilus*.

**Table 4:** pH and titratable acidity (TA), and *S. thermophilus, L. bulgaricus*,*Bifidobacterium animalis* and *Lactobacillus acidophilus* counts (log cfu/mL) of stored yogurts as a function of storage.

	Storage day					
Parameter	1	8	15	22	29	Pooled SE
pH	4.42^a^	4.34^b^	4.31^c^	4.29^c^	4.29^c^	0.01
TA (* lactic acid)	1.31^c^	1.34^b^	1.37^a^	1.39^a^	1.39^a^	0.01
*S. thermophilus*	8.54^a^	7.81^b^	7.76^b^	7.68^b^	7.53^b^	0.11
*L. bulgaricus*	8.58^a^	7.78^b^	7.03^c^	6.65^d^	5.93^e^	0.12
*B. animalis*	6.85^a^	5.91^b^	5.57^bc^	5.14^cd^	4.96^d^	0.15
*L. acidophilus*	8.63^a^	7.85^b^	7.19^c^	6.71^d^	5.75^e^	0.13

Means (*n* = 27; average for formula and culture) with different superscripts within a row are different (*P *≤* *0.05).

### Microbial counts

*Streptococcus thermophilus* counts in yogurt were not affected by formula or culture; however, the counts were significantly affected by storage (Table1). Mani-López et al. (2014) also reported that *S. thermophilus* counts in probiotic yogurt were not affected by the presence of probiotic bacteria (*L. acidophilus, L. casei or L. reuteri*). *S. thermophilus* counts in all yogurts decreased from day 1 (8.54 log cfu/mL) through day 8 (7.81 log cfu/mL), and then remained constant until day 29 (7.53 cfu log/mL; Table4). The *S. thermophilus* counts in all yogurts remained >6 log cfu/mL throughout storage. These results are consistent with Michael et al. (2010); who reported that *S. thermophilus* counts were not affected by 0.5* PE supplementation, as the counts were similar to NS yogurt throughout the storage. Thus *S. thermophilus* counts are not the limiting factor for the “Live & Active Cultures” seal.

**Figure 3:**
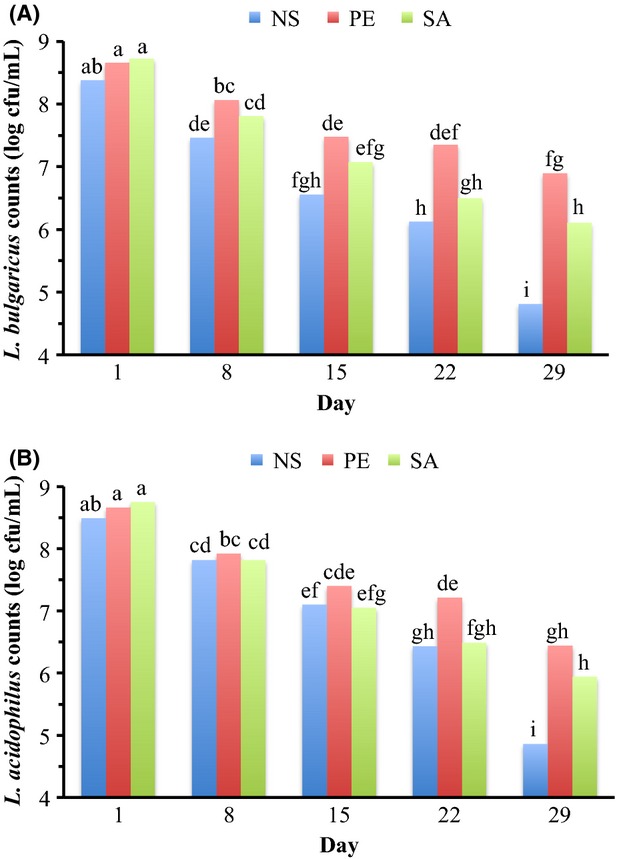
*Lactobacillus bulgaricus* counts (A) and *Lactobacillus acidophilus* counts (B) of stored yogurts^x^ as a function of formula × storage. (A): means (*N* = 9) averaged for culture with pooled standard error of 0.19; (B): means (*N* = 9) averaged for culture with pooled standard error OF 0.22. NS, non-supplemented yogurts; PE, plant extract supplemented yogurts; SA, sodium acetate supplemented yogurts. Bars with different letters are different (*P *≤* *0.05)

**Figure 4:**
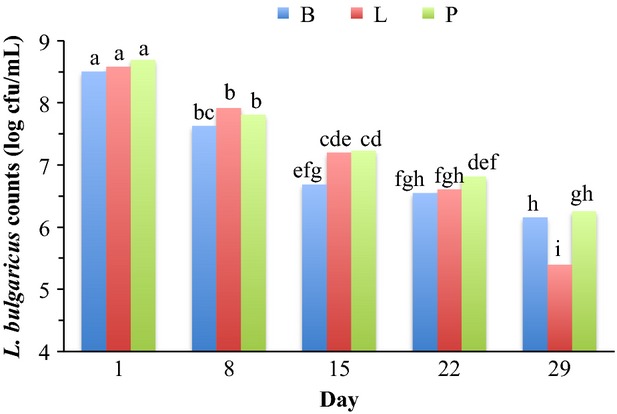
*Lactobacillus bulgaricus* counts of stored yogurts^x^ as a function of culture × storage. Means (*N* = 9) averaged for formula with pooled standard error of 0.19. B, yogurts fermented with *Bifidobacterium animalis*; L, yogurts fermented with *Lactobacillus acidophilus*; P, yogurts fermented with *B. animalis* and *L. acidophilus*. Bars with different letters are different (*P *≤* *0.05).

*Lactobacillus bulgaricus* counts were significantly affected by formula, storage, formula × culture, formula × storage, and culture × storage (Table1). *Lactobacillus bulgaricus* counts were highest in PE yogurts (7.68 log cfu/mL) followed by SA yogurts (7.24 log cfu/mL) and NS yogurts (6.66 log cfu/mL; Table2). Overall, *L. bulgaricus* counts during storage significantly decreased from day 1 (8.58 log cfu/mL) through day 29 (5.93 log cfu/mL; Table4). PE yogurt fermented with B culture had the highest *L. bulgaricus* counts (8.01 log cfu/mL) while the lowest counts were in NS yogurt fermented with B culture (6.18 log cfu/mL; Fig.2B). *Lactobacillus bulgaricus* counts within NS, PE or SA yogurts fermented with different cultures were similar; except, the counts within NS yogurts fermented with P culture were greater than yogurts fermented with B or L culture (Fig.2B). *Lactobacillus bulgaricus* counts in NS, PE or SA yogurts (8.37, 8.65 or 8.72 log cfu/mL, respectively) fermented with different cultures were similar on day 1 (Fig.3A). During storage, *L. bulgaricus* counts in NS, PE or SA yogurts significantly decreased by day 29 compared with day 1. *Lactobacillus bulgaricus* counts in PE and SA yogurts were >6 log cfu/mL throughout the storage; however, in NS yogurts the counts decreased to 4.81 log cfu/mL on day 29 (Fig.3A). *Lactobacillus bulgaricus* counts in yogurts fermented with B, L or P culture (8.49, 8.57 or 8.68 log cfu/mL, respectively) using different formula were similar on day 1 (Fig.4). During storage *L. bulgaricus* counts in yogurts fermented with B, L or P culture decreased significantly on day 29 compared with day 1. However, *L. bulgaricus* counts in yogurts fermented with B or P culture were similar and >6 log cfu/mL throughout the storage, but the counts in yogurts fermented with L culture decreased to 5.39 log cfu/mL on day 29 (Fig.4).

*Lactobacillus acidophilus* counts were significantly affected by formula, storage, and formula × storage (Table1). *Lactobacillus acidophilus* counts in PE yogurts (7.52 log cfu/mL) were greater than NS yogurts (6.94 log cfu/mL), but similar to SA yogurts (7.21 log cfu/mL; Table2). Overall, *L. acidophilus* counts decreased significantly throughout the storage from 8.63 log cfu/mL (on day 1) to 5.75 log cfu/mL (on day 29; Table4). *Lactobacillus acidophilus* counts in NS, PE or SA yogurts (8.49, 8.66 or 8.75 log cfu/mL, respectively) were similar on day 1 (Fig.3B). *Lactobacillus acidophilus* counts in yogurts with different formulation significantly decreased on day 29 compared with day 1. *Lactobacillus acidophilus* counts in PE yogurts were >6 log cfu/mL throughout the storage (Fig.3B). Although *L. acidophilus* counts in PE and SA yogurts were similar on day 29, the counts in SA yogurts were <6 log cfu/mL (5.94 log cfu/mL; Fig.3B). In NS yogurts, *L. acidophilus* counts deceased to 4.86 log cfu/mL on day 29 (Fig.3B).

The greater *L. bulgaricus* and *L. acidophilus* counts in supplemented yogurts on day 29 compared with NS yogurts (Fig.3) could be attributed to the greater buffering capacities of supplemented yogurts. Zare et al. (2011) reported that buffering capacity is a vital parameter in the growth of yogurt cultures during the yogurt fermentation; however, nutrients in the yogurt mix also influence the growth. Zare et al. (2012) reported the similar results for *Lactobacillus rhamnosus* AD 200 in probiotic fermented milk (fermented milks supplemented with 1–3* skim milk powder had greater buffering capacity and *Lactobacillus rhamnosus* AD 200 counts compared with the non-supplemented fermented milk during 28 days of storage). Greater *L. bulgaricus* counts in yogurts fermented with P culture (Fig.4) could be attributed to the synergetic effect of probiotic bacteria with *L. bulgaricus*, and improved proteolytic activity that could have provided more amino acids required for sustaining the viability of *L. bulgaricus* (Shihata and Shah 2000; Donkor et al. 2006; Mortazavian et al. 2006).

*Bifidobacterium animalis* counts were significantly affected by formula and storage (Table1). *Bifidobacterium animalis* counts in SA yogurts (6.05 log cfu/mL) were greater than PE yogurts (5.34 log cfu/mL); however, there were no significant differences between PE or SA yogurts when compared to NS yogurts (5.67 log cfu/mL; Table2). On day 1, *B. animalis* counts in yogurts were 6.85 log cfu/mL; however, the counts decreased <6 log cfu/mL on day 8 (5.91 log cfu/mL; Table4). At the end of the storage, *B. animalis* counts decreased to 4.96 log cfu/mL (Table4). Overall, the significant decrease in yogurt pH and increase in yogurt TA by day 8 could have contributed to the rapid decrease in *B. animalis* counts because *Bifidobacterium* spp. is less acid tolerant than *Lactobacillus* spp. in yogurt (Lourens-Hattingh and Viljoen 2001).

## Conclusions

Yogurts supplemented with PE or SA had greater buffering ability, and maintained greater *L. bulgaricus* and *L. acidophilus* counts by the end of the storage compared with NS yogurts. Therefore, yogurt mixes demonstrating greater buffering capacity can enhance the longevity of *Lactobacillus* bacteria in yogurt; hence, increase the shelf life of probiotic yogurt (defined as viable culture concentrations ≥6 log cfu/g). However, no significant effects of greater buffering ability of yogurt mixes were observed for *S. thermophilus* and *B. animalis*. Further research should be done to study the effect of PE supplementation on the sensory attributes of the yogurt.

## References

[b1] Ainaz A, Ehsani MR (2008). Probiotic survival in yogurt made from ultrafiltered skim milk during refrigeration storage. Res. J. Biol. Sci.

[b2] Chandan RC, Chandan RC, O'rell KR (2006). Yogurt plant: quality assurance. Manufacturing yogurt and fermented milks.

[b3] Dave RI, Shah NP (1996). Evaluation of media for selective enumeration of *Streptococcus thermophilus, Lactobacillus delbrueckii* ssp. *bulgaricus, Lactobacillus acidophilus*, and Bifidobacteria. J. Dairy Sci.

[b4] Dave RI, Shah NP (1997a). Effectiveness of ascorbic acid as an oxygen scavenger in improving viability of probiotic bacteria in yoghurts made with commercial starter cultures. Int. Dairy J.

[b5] Dave RI, Shah NP (1997b). Viability of yoghurt and probiotic bacteria in yoghurts made from commercial starter cultures. Int. Dairy J.

[b6] Donkor ON, Henriksson A, Vasiljevic T, Shah NP (2006). Effect of acidification on the activity of probiotics in yoghurt during cold storage. Int. Dairy J.

[b7] Gastaldi E, Lagaude A, Marchesseau S, de la Fuente BT (1997). Acid milk gel formation as affected by total solids content. J. Food Sci.

[b8] Heller L (2007). http://www.foodnavigator-usa.com/Business/IFT-Cognis-launches-bakery-emulsifiers-mold-inhibitor.

[b9] Lindsay RC, Damodaran S, Parkin KL, Fennema OR (2007). Food additives. Food chemistry.

[b10] Lino T, Manome A, Okada S, Uchimura T, Komagata K (2001). Effects of sodium acetate on the production of stereoisomers of lactic acid by *Lactobacillus sakei* and other lactic acid bacteria. J. Gen. Appl. Microbiol.

[b11] Lino T, Uchimura T, Komagata K (2002). The effect of sodium acetate on the growth yield, the production of L- and D-lactic acid, and the activity of some enzymes of the glycolytic pathway of *Lactobacillus sakei* NRIC 1071^T^ and *Lactobacillus plantarum* NRIC1067^T^. J. Gen. Appl. Microbiol.

[b12] Lourens-Hattingh A, Viljoen BC (2001). Yogurt as probiotic carrier food. Int. Dairy J.

[b13] Lucey JA, Gorry C, Fox PF (1993a). Acid-base buffering properties of heated milk. Milchwissenschaft.

[b14] Lucey JA, Hauth B, Gorry C, Fox PF (1993b). The acid-base buffering properties of milk. Milchwissenschaft.

[b15] Mani-López E, Palou E, López-Malo A (2014). Probiotic viability and storage stability of yogurts and fermented milks prepared with several mixtures of lactic acid bacteria. J. Dairy Sci.

[b16] Manju S, Jose L, Gopal TKS, Ravishankar CN, Lalitha KV (2007). Effects of sodium acetate dip treatment and vacuum-packaging on chemical, microbiological, textural and sensory changes of pearspot (*Etroplus suratensis*) during chill storage. Food Chem.

[b17] Michael M, Phebus RK, Schmidt KA (2010). Impact of a plant extract on the viability of *Lactobacillus delbrueckii* ssp. *bulgaricus* and *Streptococcus thermophilus* in nonfat yogurt. Int. Diary J.

[b18] Moriya J, Fachin L, Gândara ALN, Viotto WH (2006). Evaluation of culture media for counts of *Bifidobacterium animalis* in the presence of yoghurt bacteria. Braz. J. Microbiol.

[b19] Mortazavian AM, Ehsani MR, Mousavi SM, Sohrabcvandi S, Reinheimer JA (2006). Combined effect of temperature-related variables on the viability of probiotic microorganisms in yogurt. Aust. J. Dairy Sci.

[b20] NYA (National Yogurt Association) (2012). http://aboutyogurt.com/.

[b21] Ross RP, Desmond C, Fitzgerald GF, Stanton C (2005). Overcoming the technology hurdles in the development of probiotic foods. J. Appl. Microbiol.

[b22] Ruzin SE (1999). Buffers. Plant microtechnique and microscopy.

[b23] Salaün F, Mietton B, Gaucheron F (2007). Influence of mineral environment on the buffering capacity of casein micelles. Milchwissenschaft.

[b24] Sarkar S (2008). Effect of probiotics on biotechnological characteristics of yoghurt – a review. Br. Food J.

[b25] Shafiee G, Mortazavian AM, Mohammadifar MA, Koushki MR, Mohammadi A, Mohammadi R (2010). Combined effect of dry matter content, incubation temperature and final pH of fermentation on biochemical and microbiological characteristics of probiotic fermented milk. Afr. J. Microbiol. Res.

[b26] Shah NP, Lankaputhra WEV, Britz ML, Kyle WSA (1995). Survival of *Lactobacillus acidophilus* and *Bifidobacterium bifidum* in commercial yoghurt during refrigerated storage. Int. Dairy J.

[b27] Shihata A, Shah NP (2000). Proteolytic profile of yogurt and probiotic bacteria. Int. Dairy J.

[b28] van Slyke DD (1922). On the measurement of buffer values and on the relationship of buffer value to the dissociation constant of the buffer and the concentration and the reaction of the buffer solution. J. Biol. Chem.

[b29] Stanton C, Gardiner G, Meehan H, Collins K, Fitzgerald G, Lynch PB (2001). Market potential for probiotics. Am. J. Clin. Nutr.

[b30] Vasiljevic T, Shah NP (2008). Probiotics – from Metchnikoff to bioactives. Int. Dairy J.

[b31] Vasiljevic T, Kealy T, Mishra VK (2007). Effect of *β*-glucan addition to a probiotic containing yogurt. J. Food Sci.

[b32] Zare F, Boye JI, Orsat V, Champagne C, Simpson BK (2011). Microbial, physical and sensory properties of yogurt supplemented lentil flour. Food Res. Int.

[b33] Zare F, Orsat V, Champagne C, Simpson BK, Boye JI (2012). Microbial and physical properties of probiotic fermented milk supplemented with lentil flour. J. Food Res.

